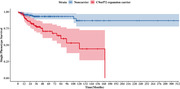# C9orf72 repeat expansions increase risk for secondary symptom development in amyotrophic lateral sclerosis and behavioral‐variant frontotemporal degeneration

**DOI:** 10.1002/alz.090459

**Published:** 2025-01-03

**Authors:** Barbara E Spencer, Sharon X Xie, Lauren Elman, Colin Quinn, Defne A Amado, Michael Baer, Eddie B Lee, Vivianna M Van Deerlin, Laynie Dratch, Lauren Massimo, David J Irwin, Corey T McMillan

**Affiliations:** ^1^ University of Pennsylvania, Philadelphia, PA USA; ^2^ University of Pennsylvania, School of Nursing, Philadelphia, PA USA; ^3^ Department of Neurology, Perelman School of Medicine, University of Pennsylvania, Philadelphia, PA USA

## Abstract

**Background:**

In amyotrophic lateral sclerosis and behavioral‐variant frontotemporal degeneration, the presence of secondary cognitive‐behavioral or motor symptoms, respectively, is associated with shorter survival. However, factors influencing the risk of secondary symptom development remain largely unexplored and time‐to‐event characterization for developing secondary symptoms is important for prognostic clinical decision‐making.

**Method:**

We performed a retrospective evaluation of the entire disease course of individuals in the Penn Integrated Neurodegenerative Disease Database with a primary clinical phenotype of amyotrophic lateral sclerosis (n = 173) or behavioral‐variant frontotemporal degeneration (n = 69). All individuals were evaluated for *C9orf72* expansions (>30 repeats). Only individuals who had neuropathological confirmation of a TDP‐43 proteinopathy at autopsy or had a *C9orf72* repeat expansion were included for analysis. We examined the odds and hazard of secondary symptom development and assessed whether they were modified by the presence of a *C9orf72* repeat expansion or primary clinical phenotype.

**Result:**

Binary logistic regression and Cox proportional hazard analyses revealed increased odds (odds ratio = 4.26 [1.98‐9.18]; p<0.001) and an increased hazard (hazard ratio = 4.79 [2.33‐ 9.82], p<0.001) for developing secondary symptoms in *C9orf72* expansion carriers compared to noncarriers. Primary phenotype, age at symptom onset, and sex were not associated with risk for secondary symptom development.

**Conclusion:**

These findings highlight the need for clinician vigilance to detect the onset of secondary cognitive‐behavioral and motor symptoms in patients carrying a *C9orf72* repeat expansion, regardless of primary phenotype, and may warrant dual referrals between cognitive and neuromuscular clinics in these cases.